# Beyond sequence homology: Cellular biology limits the potential of *XIST* to act as a miRNA sponge

**DOI:** 10.1371/journal.pone.0221371

**Published:** 2019-08-16

**Authors:** Erin A. Marshall, Greg L. Stewart, Adam P. Sage, Wan L. Lam, Carolyn J. Brown

**Affiliations:** 1 Department of Integrative Oncology, BC Cancer Research Centre, Vancouver, Canada; 2 Interdisciplinary Oncology Program, University of British Columbia, Vancouver, Canada; 3 Department of Medical Genetics, University of British Columbia, Vancouver, Canada; University of Toronto, CANADA

## Abstract

**Introduction:**

The sponging of microRNAs by a long non-coding RNA (lncRNA) away from their coding gene targets is a conceptually-simple, yet biologically-complex method of lncRNA-mediated gene regulation. Currently, predictions of genes that participate in sponge-based regulation are largely based on sequence homology alone, which may not adequately reflect the cellular environment in which lncRNA:miRNA pairs interact. The vast number of potential interactions generated by these predictions impedes the identification of functional gene regulatory relationships, which necessitates an approach that considers biological context. *XIST*, the female-specific lncRNA canonically involved in silencing the X chromosome, has been suggested by many studies to act as a miRNA sponge. The sex-specificity of *XIST* provides the opportunity to study the biological feasibility of proposed *XIST*-miRNA interactions. Here we take a comprehensive approach by considering factors that affect possible regulation through *XIST*-miRNA sponging.

**Results:**

To identify the most feasible candidates in a particular tissue (lung adenocarcinomas), we considered protein-coding genes that (1) were positively correlated with *XIST* expression within sexes, (2) were targeted by miRNAs shared with *XIST*, and (3) expressed in lung adenocarcinoma. This revealed a robust set of 124 genes potentially positively regulated by *XIST* through the sequestration of 804 shared miRNAs. We then used the basic sex-specific nature of *XIST* to compare the changes in miRNA-target gene relationships in endogenously high-*XIST* and low-*XIST* systems to discover a high-confidence set of only 13 miRNA-gene pairs. As *XIST* is expressed exclusively in the nucleus, we validated the nuclear presence of several of these high-confidence miRNAs using RT-qPCR, confirming the co-localization required for *XIST* to interact with these species.

**Conclusions:**

We use a biology-driven approach to identify genes defended from miRNA-based inhibition by the lncRNA *XIST*. Importantly, we identify that only a small subset of miRNAs predicted by sequence homology alone have the capacity to mediate the *XIST*-target gene axis, as they are enriched in the nucleus and able to co-localize with *XIST* for sponging. Our results reinforce the necessary consideration of biological features in future studies of lncRNA:miRNA interactions.

## Introduction

**Fig 1:**
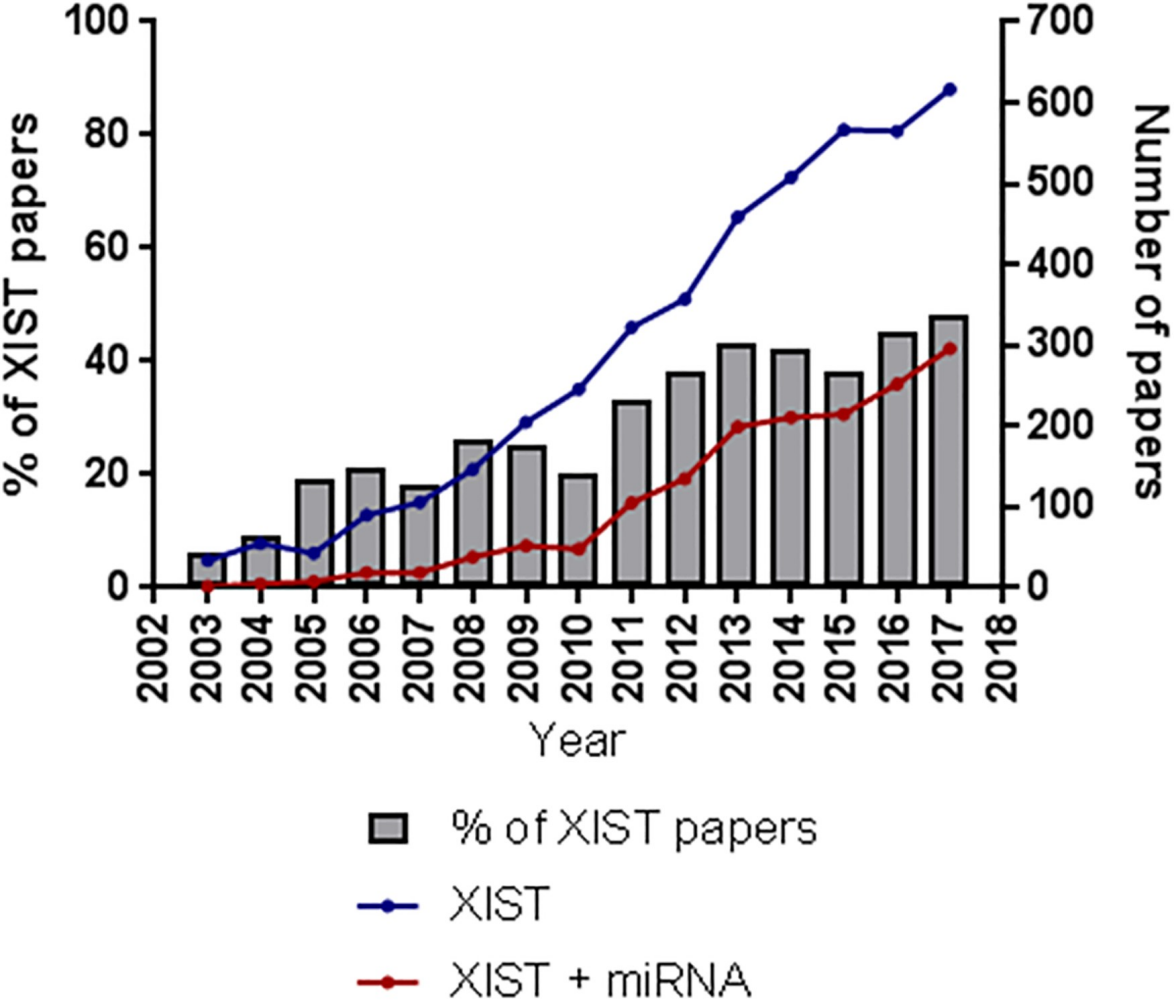
Literature concerning *XIST* biology is dominated by miRNA associations. Number of PMC results per year using “*XIST*” (blue) or “*XIST* AND miRNA” (red) as search terms.

Long non-coding RNAs (lncRNAs; >200nt) are recognized as crucial mediators of gene expression [1–3]. With the rapidly increasing number of lncRNAs identified, diverse mechanisms-of-action are being discovered. A type of non-coding RNA mediated regulation known as “miRNA sponging” has been cited in over 600 publications in the last year, a number that has been exponentially increasing over the past decade and has come to dominate lncRNA literature (Fig 1, S1 Fig). MiRNAs are small (~22 nt), non-coding transcripts that negatively regulate gene expression through direct base-pairing with coding mRNAs in regions of as little as six nucleotides [4,5]. However, a miRNA may interact with multiple target genes irrespective of their coding capability [6]. In theory, a lncRNA sponge acts as a decoy in the cell, positively regulating coding mRNAs that harbour the same miRNA target sequence by binding with the shared inhibitory miRNAs, thereby decreasing the abundance of the miRNA species [7–9]. Correspondingly, most RNA transcripts, particularly lncRNA sponges, harbour multiple miRNA target sites, and thus have the potential to be regulated by multiple miRNA rather than simply a single species, with interactions governed by biological context [10].

*XIST* was one of the first functionally-characterized lncRNAs, and is canonically involved in *cis*-silencing of an X chromosome in females, a mechanism of dosage compensation that prevents an imbalance in X-linked gene expression between males and females [11–13]. There has been growing recognition of a potential role for the *XIST*-coated inactivated X chromosome in cancer [10,14]; however, the proposed oncogenic mechanism of *XIST* remains contentious, a problem exacerbated by conflicting mechanistic reports that differ depending on the cancer type and cancer-associated phenotype probed. In recent years, a significant fraction of the literature on *XIST* has been devoted to the implication of its function as a miRNA sponge (Fig 1). Many of these studies contemplated the involvement of *XIST* in protecting cancer-related genes from miRNA-mediated negative regulation. While intriguing, these studies often focus on the interaction of *XIST* with one miRNA shared with one target gene; however, the number of binding sites on the >17 kb mature human *XIST* RNA suggests a more complex potential landscape of shared miRNAs and target genes. Further, while many current *ex vivo* assays can confirm miRNA binding, they do not reflect the intricacies of a biological system with multiple target genes, highlighting the need for an investigation to determine which mRNAs are most efficiently regulated by *XIST* in this manner.

Here, we take a comprehensive, biologically-relevant approach by considering factors that affect preferential regulation by miRNA sponging, revealing a significantly-reduced yet robust set of genes potentially positively regulated by *XIST* through the sequestration of miRNAs (for brevity, we call these target genes defended from miRNA by *XIST*, DMX genes). Using *XIST*, we explore features that need to be taken into account when studying the effects of miRNA sponging.

## Results

### Samples

While *XIST* expression is expected in all female somatic cells, we chose to interrogate lung adenocarcinoma (LUAD). LUAD occurs in both sexes, and thus allows for the assessment of high-*XIST* (female, n = 304) and low-*XIST* (male, n = 264) systems. Additionally, normal lung tissues present with a large range range of *XIST* expression in females, allowing for increased power and observed strength of correlation (red arrow, S2 Fig) [15].

### Idenification of genes defended from miRNA by *XIST* (DMX genes) in female lung adenocarcinomas

**Fig 2:**
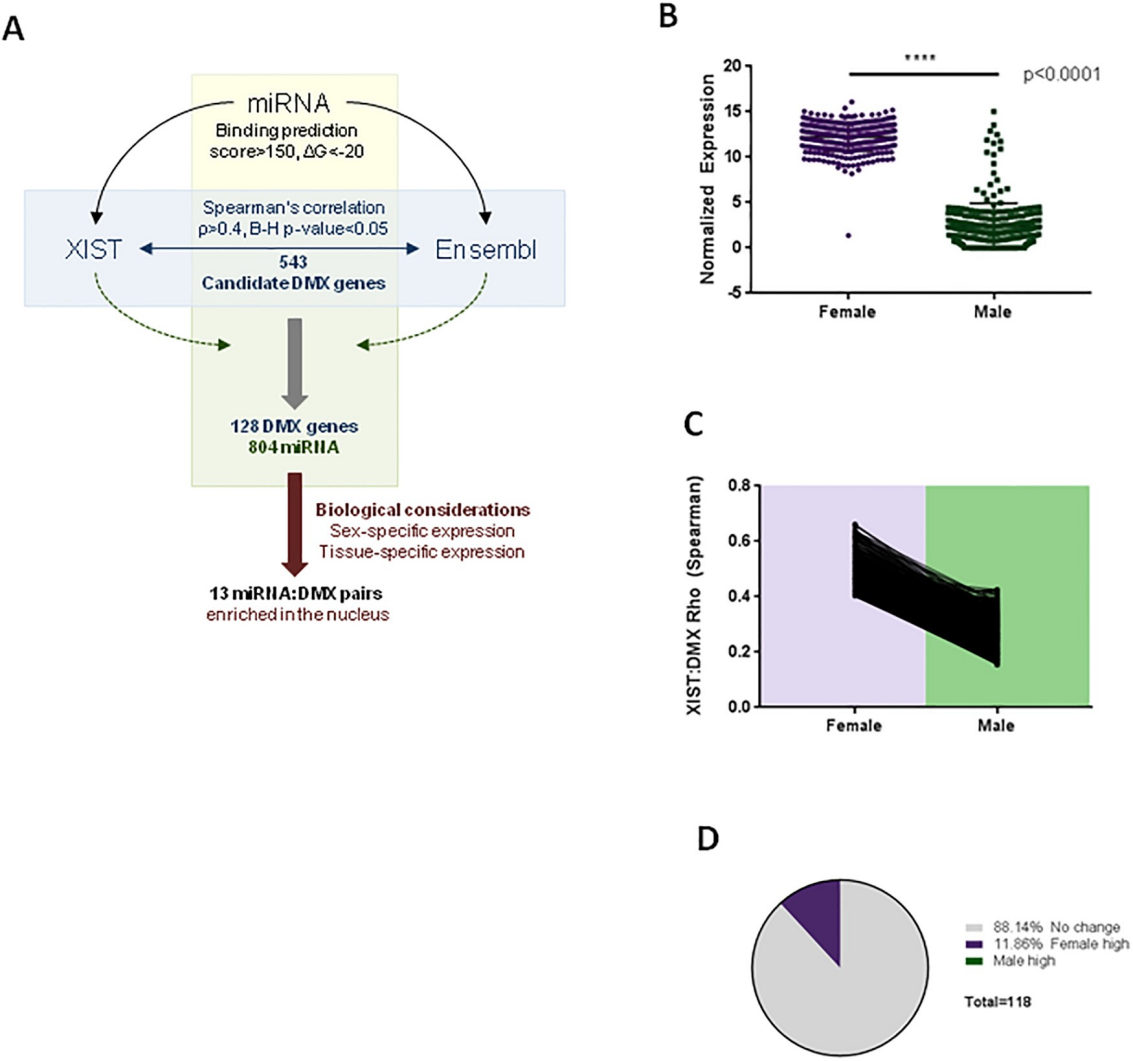
*XIST* is increased in females and regulates DMX genes. A) Flow chart for the identification of DMX genes and biologically-relevant sponged miRNAs. B) Expression of *XIST* is significantly elevated in females (purple) compared to males (green). C) *XIST*-DMX gene expression relationships are decreased in males compared to females (Spearman’s Rho, B-H p≤0.05 in females). D) Proportion of DMX genes (n = 118) significantly differentially expressed between sexes (Student’s t-test, p≤0.05).

A summary of the analysis pipeline is shown in Fig 2A. Unsurprisingly, in lung adenocarcinoma sequencing data, *XIST* was observed to be expressed at higher levels in female tumours when compared to male tumours (n = 235) (Student’s t-test, p<0.0001; Fig 2B). As miRNA sponges positively regulate their target genes, we first sought to identify genes that could be affected by changes in *XIST* expression. In female LUAD, expression of all Ensembl-annotated genes was compared with *XIST* expression to identify positive correlations. In total, 543 candidate genes met our minimum threshold (Spearman’s Rho>0.4, B-H p≤0.05) for association with *XIST* expression. This initial query was strictly performed in females, as females present with a normal range of *XIST* trancript expression, and assessment in a mixed sex sample would conflate unrelated sex differences with potential sponging effects. In the same manner, this relationship was assessed in male LUAD samples. In males compared to females, these same 543 relationships were significantly decreased to below our detection threshold (Fig 2C).

To assess the potential of miRNAs to bind both their target genes and *XIST* (as the proposed sponge), the 3’ untranslated regions (3’ UTR) of these 543 candidate DMX genes were retrieved from the UCSC Genome Browser, and all annotated miRNAs were assessed for binding against these DMX genes using the miRanda binding algorithm (ΔG≥-20kCal/mol, score>150). A total of 10,654 miRNA/DMX gene pairs were predicted, which includes 2,052 unique miRNA targeting 124 unique DMX genes. This algorithm was run in an unbiased manner against the complete (unspliced) *XIST* sequence and all annotated miRNAs with the same energy and score thresholds. In total, 864 miRNAs were predicted to bind *XIST* (S2 Table), while 804 unique miRNAs were predicted to target both *XIST* and at least one of 124 unique DMX genes (S3 Table). In order to be sponged by a lncRNA, these miRNAs must be expressed in the same tissue. As miRNAs are often tissue specific, we confirmed the expression of candidate miRNAs in lung tumour tissue. Of the 124 DMX genes, 11.86% were significantly more expressed in female compared to male-derived tumours (Student’s t-test, p<0.05), suggesting a potential sex-specific mechanism of *XIST*-mediated gene regulation in lung tumours (Fig 2D, S4 Table).

### miRNAs targeting *XIST* exonic regions display stronger DMX relationships

**Fig 3:**
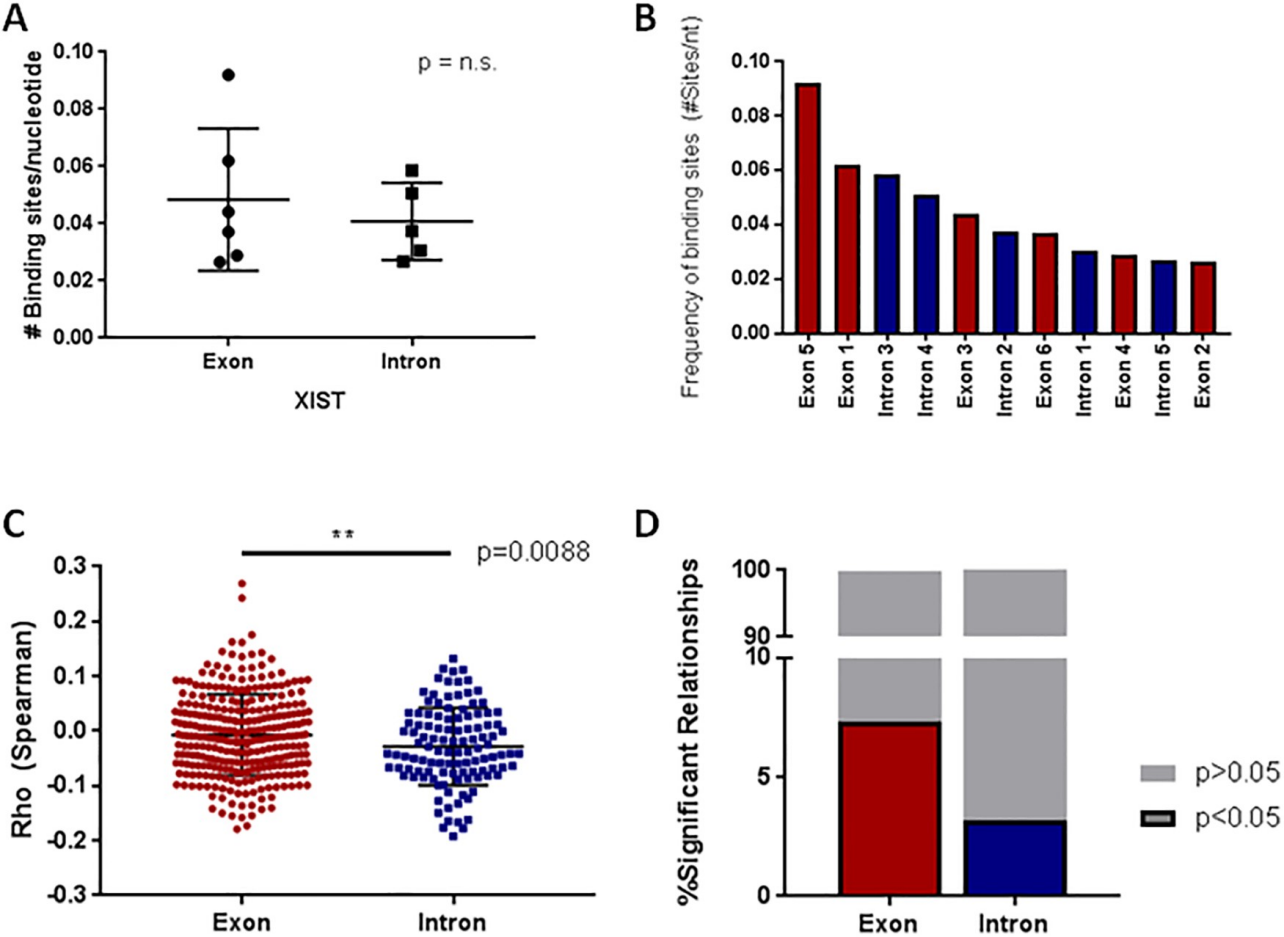
miRNAs exonically-bound to *XIST* exhibit increased relationships with DMX genes. A) The number of miRNA binding sites on *XIST* is not enriched when introns and exons are compared. B) Number of miRNA binding sites on the introns and exons of *XIST*; exon 5 has the greatest concentration of miRNA binding sites. C) miRNAs bound to *XIST* exclusively on exons exhibit stronger miRNA-DMX Spearman’s correlation values. D) Similarly, a larger proportion of correlations between miRNA-DMX pairs are significant when the miRNA binds to an exonic (vs. intronic) region of *XIST*. In this Fig, exons are depected in red, while introns are depicted in blue.

We aimed to determine whether the region of miRNA binding on the *XIST* transcript, and thus splicing, had an affect on their ability to bind DMX genes. Positional information of the 804 miRNAs predicted to bind *XIST* and candidate DMX genes was aligned to the *XIST* transcript and mapped relative to the transcript exonic boundaries (S3 Fig and S5 Table). While there was no particular enrichment for number of binding sites in intronic or exonic regions overall (Student’s t-test, p = n.s., Fig 3A), exon 5 exhibited a marked enrichment in number of miRNA binding sites relative to its size (15 sites within 163 nucleotides; Fig 3B). Interestingly, miRNAs binding in only exonic regions of *XIST* exhibited stronger magnitudes of correlation with their corresponding DMX genes than those binding in intronic regions (Fig 3C). Similarly, a greater number of miRNA-DMX gene relationships reached the threshold of significance when the miRNAs targeted only exonic regions (Fig 3D). These findings are particularly relevant in biological contexts as the fully-spliced *XIST* transcript is the highest-abundance *XIST* transcript in normal lung tissues, and canonically functions in the nucleus [15] (S4 Fig). Together, our results suggest that exonic regions of *XIST* may be the most available for miRNA binding and thus sponge-based gene regulation.

Transcriptome-wide, *XIST* is not enriched in number of miRNA binding sites compared to other lncRNAs by length (S5A Fig). Similarly, *MALAT1* and *NEAT1*, other commonly-studied lncRNAs frequently reported to act as miRNA sponges in cancer, are similarly not enriched in number of binding sites. When we examined the number of target sites for each miRNA on the *XIST* transcript, many had multiple binding sites, similar to the requirements of artificial sponging optimization [7,16] (S5B and S5C Fig). Interestingly, DMX genes with a greater number of shared miRNA binding sites with *XIST* exhibit stronger *XIST*-DMX relationships, suggesting that this regulatory axis is likely mediated by a pool of miRNAs rather than a single species (S5D and S5E Fig). Interestingly, while XIST:DMX correlations generally get stronger in samples with increasing miRNA expression, samples with the highest miRNA expression levels show a decrease in correlation. This may suggest that, in some cases, high miRNA expression levels may be able to quench XIST, reducing its ability to effectively sponge miRNAs. Ranked lists of miRNAs predicting binding of both putative sponge and target can be generated for other candidate lncRNA sponges; however, the sex-specific nature of *XIST* allows another step towards examining the biological feasibility of the sponge interaction.

### Systems with high-*XIST* reveal nuclear-enriched subset of miRNAs that mediate the *XIST*-DMX axis

**Fig 4:**
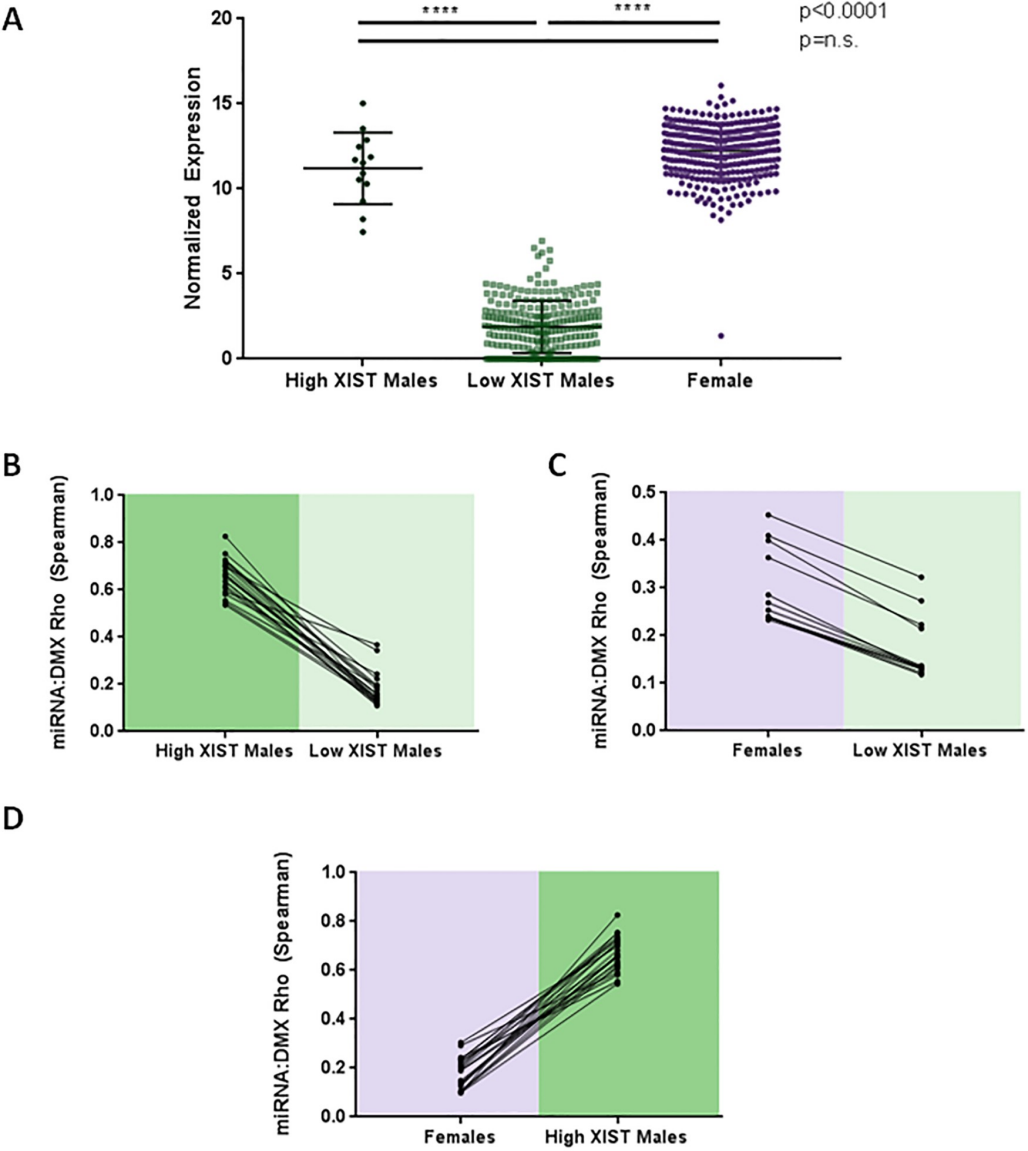
Males with High-*XIST* display female-like patterns. A) A subset of males have *XIST* expression equivalent to female expression levels (High-*XIST* males). B) When compared to Low-*XIST* males, High-*XIST* males show significantly stronger relationships between the candidate miRNA and DMX gene. C) Similarly, females display significantly stronger relationships between the candidate miRNA and corresponding DMX gene than Low-*XIST* males (change in Spearman’s coefficient of 0.1 or greater shown). D) When miRNA-DMX releationships are compared in High-*XIST* systems, males had significantly stronger correlation coefficients than females with equivalent levels of *XIST*.

As we expected that sponging interactions would be specific to females (as they express the *XIST* transcript), we first assessed which miRNA-DMX gene relationships were most significantly altered in high-*XIST* compared to low-*XIST* tumours. As some males exhibited higher-than-expected *XIST* expression, we separated the male LUAD cohort into low-*XIST* (mean expression ± 2 standard deviations) from those that had expression that exceeded this range (high-*XIST* males). To rule out mis-classified female cases, Y-gene expression was confirmed in both high and low-*XIST* male samples. We observed that the average number of reads from the Y chromosome from the Low *XIST* and High *XIST* males were equivalent (p = n.s.) and significantly greater than those from the female patients (p<0.0001) (Student’s t-test, S6A Fig). The high-*XIST* male-derived tumours exhibited female-level *XIST* expression (Student’s t-test, p = n.s., Fig 4A), and both male and female high-*XIST* systems had greater expression of *XIST* than low-*XIST* males (Student’s t-test, p<0.0001; Fig 4A). The miRNA-DMX gene relationships in high-*XIST* systems (both male and female) were compared to those in the low-*XIST* systems, and the strength of significant correlations in the high-*XIST* systems was assessed relative to the low-*XIST* system. While we would expect miRNA-DMX expression association to approach zero through canonical miRNA degradation mechanisms, we find that in the presence of *XIST*, miRNA and DMX genes display positive relationships, indicating that the action of the miRNA is inhibited (Fig 4B and 4C). Furthermore, while both high-*XIST* systems have miRNA-DMX relationships that decrease when compared to the low-*XIST* tumours, the miRNA-DMX relationships are stronger in males with elevated *XIST* (n = 12) than in females with comparable *XIST* expression (Fig 4D). To ensure that the uneven sample size was not artifically selecting for an unrepresentative subset of interactions, we compared miRNA-DMX relationships that met the threshold of significance in Low XIST and High XIST male cohorts and found that they had equivalent correlations in females (Student’s t-test, p = n.s., S6B Fig).

To determine which miRNAs may indeed be affected by the presence of *XIST*, we set a threshold of change in miRNA-DMX correlation value (ΔRho) between the high-*XIST* and low-*XIST* systems of at least -0.1. This threshold produced 13 miRNA-DMX gene pairs (Table 1). Of the 13 gene pairs identified, 5 contained miRNAs with known nuclear localization, a relatively rare phenomenon for miRNAs but common for lncRNAs such as *XIST*. Interestingly, none of the miRNAs contained the 3’ hexanucleotide sequence present on miR-29b known to result in localization of processed small RNA molecules to the nucleus (S7 Fig).

**Table 1:** miRNA and DMX relationships altered in the presence of *XIST*. miRNA and corresponding predicted target genes with accompanying Spearman’s Rho correlation coefficient and Benjimini-Hochberg p-values.

		Females		Low *XIST* Males		DIFFERENCE
miRNA	Gene	Rho	p	Rho	p	Diff. in Rho
hsa-mir-29b	CRIPAK	0.39982	0	0.21468	0.00051	-0.1851
hsa-mir-20a	PARP6	0.28577	1E-06	0.12964	0.02429	-0.1561
hsa-mir-93	LPIN3	0.36396	0	0.2232	0.00031	-0.1408
hsa-mir-30c	ING5	0.41037	0	0.27319	1.3E-05	-0.1372
hsa-mir-1254	MAPK8IP3	0.26901	2E-06	0.1368	0.01866	-0.1322
hsa-mir-660	CCDC57	0.45355	0	0.32275	0	-0.1308
hsa-mir-1301	EWSR1	0.23789	2.7E-05	0.11808	0.03632	-0.1198
hsa-mir-1301	CENPT	0.25362	8E-06	0.13482	0.02009	-0.1188
hsa-mir-106a	MDM4	0.24035	2.3E-05	0.12213	0.03165	-0.1182
hsa-mir-361	MAPK8IP3	0.2379	2.9E-05	0.12968	0.02426	-0.1082
hsa-mir-29b	PAN2	0.23928	2.6E-05	0.13406	0.02067	-0.1052
hsa-mir-361	ZBTB40	0.23309	3.9E-05	0.13106	0.02307	-0.102

**Fig 5:**
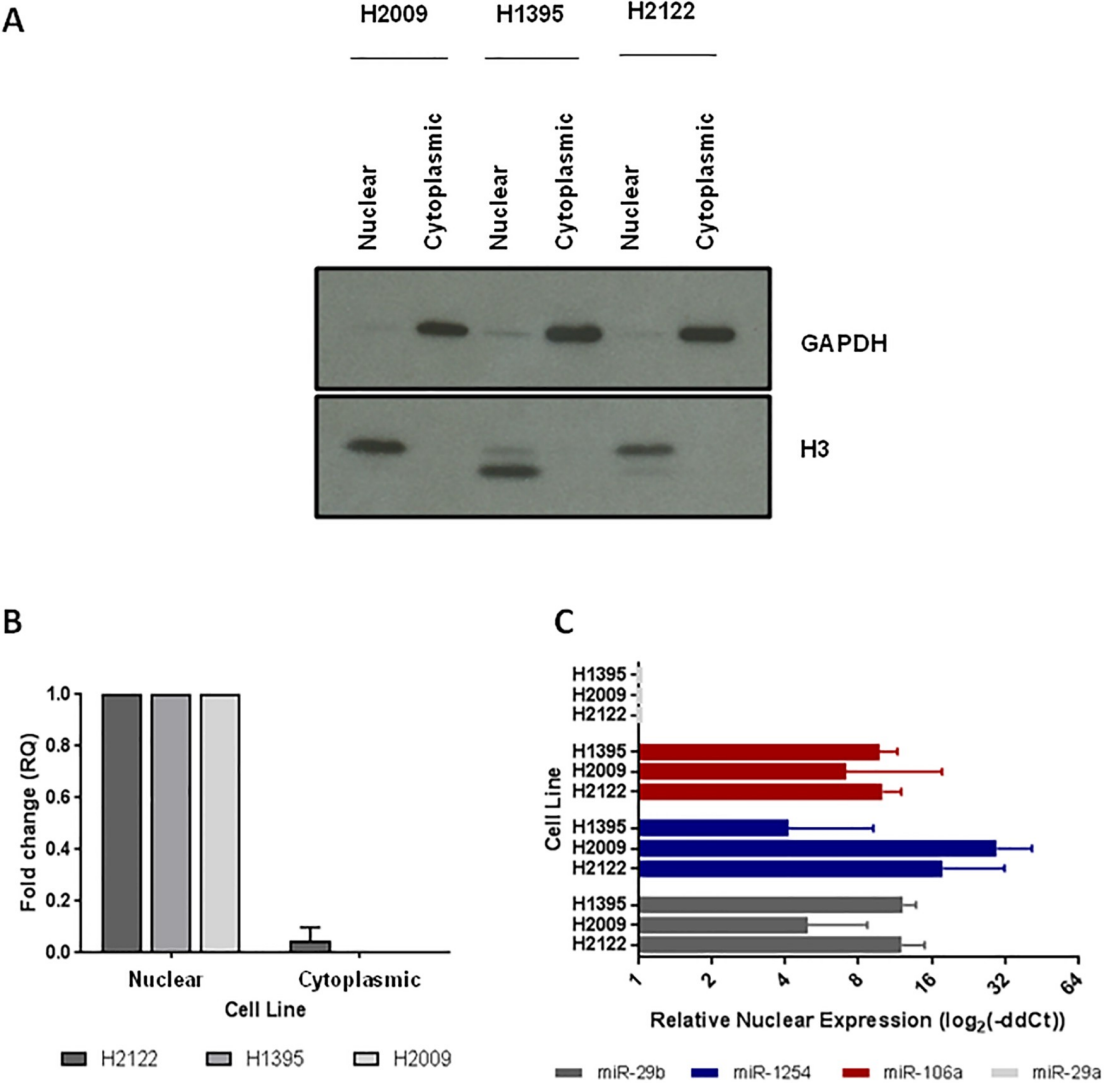
Analysis of *XIST*-expressing systems reveals *XIST*-miRNA-DMX gene axes enriched in nuclear miRNA. A) LUAD cell lines were fractionated by subcellular compartment, and the nuclear and cytoplasmic protein fractions showed strong enrichment of H3 and GAPDH, respectively (nuclear and cytoplasmic proteins). B) RT-qPCR of nuclear and cytoplasmic RNA from LUAD cell lines confirms that *XIST* is present only in the nuclear fraction. C) Similarly, the ratio of miRNA expression in the nuclear and cytoplasmic cell line fractions shows that miR-106a and miR-1254 are enriched in the nucleus at a similar level as miR-29b, a miRNA with a known nuclear localization sequence. This enrichment is not seen in miR-29a, a miRNA with known cytoplasmic localization in its mature form.

To confirm that the miRNAs produced from this pipeline were indeed nuclear, allowing for their interaction with the spliced *XIST* transcript, we validated their presence in the nucleus of female-derived lung cancer cell lines with wild-type X chromosomes. To confirm subcellular fractionation, protein lysates were harvested from the cytoplasmic and nuclear fractions of each cell line. Western blots for the cytoplasmic GAPDH and nuclear H3 verified the purity of each fraction (Representative image shown in Fig 5A). Furthermore, RT-qPCR revealed *XIST* was only present in the nucleus (and not the cytoplasm) of three LUAD cell lines, necessitating the presence of the proposed miRNAs in the nucleus if they were to interact with *XIST* (Fig 5B). We then validated the presence of 3 of 9 miRNAs in the LUAD cell line nuclear fractions by RT-qPCR. miR-29b (a known nuclear miRNA) was present at an average of 67.5%, while miRNA-106a and miR-1254 were present at 37.3% and 73.7% of the cytoplasmic fraction, respectively (Fig 5C). Given the nuclear localization of *XIST*, this pattern of miRNA subcellular localization strengthens the potential of these interactions, and increases the feasibility of sponging.

## Discussion

In this study, we aimed to determine *XIST*-miRNA-DMX relationships that are consistent with *XIST* biology. Beyond sequence homology and expression correlations with coding genes, we set out to consider relevant biological factors specific to the expression of *XIST* that may affect its sponging capacity. Using the sex-specific expression of *XIST* we have determined that putative miRNA binding sites in exons rather than introns show greater DMX:*XIST* correlations, and that the best candidates share nuclear localization with *XIST*.

Using sequence-based target prediction, we find that 804 of the 862 miRNAs that bind *XIST* are shared with DMX genes. These DMX genes are also highly correlated with *XIST* expression, and suggest that DMX gene expression could be positively regulated by *XIST* through a mechanism biologically consistent with miRNA sponging. Additionally, we observed that a subset of these DMX genes are upregulated in females, while none are upregulated in males, suggesting the importance of lncRNA sponging of miRNAs as a method of sex-specific genome regulation. The number of binding sites per miRNA on the *XIST* transcript is relevant to miRNA sponging efficiency. Optimization studies of long RNA transcripts as sponges in an experimental setting have determined that sponges with 4–10 binding sites per miRNA have the greatest effect on target gene expression, of which 10% of our candidate miRNAs fall within this range. Interestingly 5 of our 9 high confidence miRNAs had 2 or more binding sites on *XIST* [17]. Further, miRNAs with more binding sites on their target gene exhibit stronger correlations, suggesting that a pool of miRNAs may be acting as intermediaties in this axis.

*XIST* has numerous splice isoforms, and we sought to explore whether splicing had an effect on *XIST*-mediated miRNA sponging to determine if the stable nuclear-functioning spliced transcript may be acting as a sponge. The Spearman’s Correlation Rho and p values indicate that the spliced transcript has a stronger effect on DMX gene expression and interestingly, the fully-spliced transcript is the most abundant isoform in lung tissue (Fig 3B and 3C, S4A Fig) [15]. Thus, the spliced transcript is likely the primary contributor to the miRNA sponging observed in cells.

Importantly, in cellular systems that lack *XIST*, the relationship between miRNA and DMX gene expression is decreased in magnitude (Fig 4B and 4C), consistent with *XIST* acting as a miRNA sponge in the *XIST*-containing systems. When XIST is present, the sequestration of miRNAs limits degradation of the target gene and a more positive correlation between DMX and miRNA is observed. In the systems with low levels of XIST, the free miRNAs are able to exert a negative regulatory effect by binding to DMX genes and we observe that the correlation with miRNA levels is reduced. If DMX genes are regulated by *XIST*, the shared miRNAs and *XIST* must be present in the same cellular compartment, in order to interact. By considering relevant biological features, we found that only 1% of the miRNAs predicted by sequence homology alone (9 of the 864 we predict to interact with *XIST*) are likely affected by endogenous *XIST* expression. The presence of mature miRNA in the nucleus is a relatively rare observation in miRNA biology, but we were encouraged to find that these species are found in a cellular location necessary for interaction with the sponge to occur. Although *XIST* localization is known to be exclusively nuclear [11], it is possible that *XIST* may be briefly accessible to non-nuclear miRNAs for sponging during cell division, although this has not been shown. Thus, any miRNA that has the potential to be sponged by *XIST* must exist at high concentrations in the nucleus. Beyond subcellular localization, our analyses reveal the substantial effects of features such as sex- and tissue-specific expression as well as splicing isoforms on the feasibility of lncRNA:miRNA sponging interactions.

Of particular interest was the increase in miRNA-DMX relationship magnitude when comparing the two high-*XIST* systems which had equivalent average Rho values (Fig 4D, S6B Fig). We hypothesize that this increase may be due to abberant *XIST* expression from the active male X chromosome, and thus the transcript lacks the opportunity to perform its canonical X-inactivation function in males. This increase in available transcripts may allow for an elevated number of miRNAs to be sponged, resulting in a greater effect on DMX gene expression.

We then sought to confirm the presence of the best miRNA sponging candidates in the nucleus for species where literature on their cellular location did not exist. We validated the presence of miR-29b, a miRNA known to be translocated after complete processing to the nucleus because of a 3’ hexanucleotide sequence in its mature form [18]. While this nucleotide pattern is not enriched in this subset of miRNAs (S7 Fig), miR-106a and miR-1254 were both observed to be in the nucleus of the three LUAD cell lines tested (Fig 5). This may suggest that, although these miRNA lack a known nuclear localization sequence, there may be a secondary method of localization that is causing the distribution of these species back to the nucleus. Recent studies have shown preferential shuttling of partially-processed pre-miRNA complexed with RISC, however further research will be needed to determine if this mechanism is involved in miRNA sponging [19].

While *XIST* has been proposed to function as a miRNA sponge in many studies, we did not observe any of these previously described *XIST*-miRNA interactions in our high-confidence miRNA-DMX pairs. One reason for this discrepancy may be differences in tissue specific expression. We performed our analysis in LUAD, but as miRNA expression patterns are known to be tissue-specific, unique miRNA expression profiles will likely result in unique sets of miRNA-DMX pairs with tissue-relevant biological implications [17,20,21]. Similarly, it is worth noting that even in scenarios where miRNA and *XIST* expression levels are constant across tissues, the interactions most important to cellular biology will likely change due to the tissue-specific gene expression patterns of coding-gene targets. Another important consideration is the size and complexity of miRNA-sponging networks. MiRNAs target hundreds of genes, and mRNA targets often contain many miRNA binding sites. Adding to this complexity is the fact that many lncRNAs are targeted by hundreds of miRNAs (for example, we predicted 864 miRNAs to interact with *XIST* by sequence homology alone), potentially leading to sponging networks with thousands of interactions. Additionally, proving the effect of these proposed complex interactions is difficult: assays confirming a miRNA can bind to both sponge and target (for example, luciferase assays) are important, but function in simplified systems that do not approximate normal cell processes. The network of genes that interact with a single miRNA is complex, and when considering sponges that bind multiple microRNA, this complexity is further amplified. These networks suggest that it is unlikely that one miRNA is being prefentially sequestered and affecting one target gene to mediate a cancer phenotype. Finally, when changing the endogenous expression of a sponge or miRNA in cell models, one must consider the potential for off-target affects due to the vast number of predicted targets that are present in any sponging network.

As a sex-specific lncRNA, *XIST* presents the opportunity to study human cancer cells from tumours with dichotomous *XIST* expression and determine how the presence of this transcript in the system could be causing downstream gene expression changes. Of the 864 miRNA predicted to bind *XIST* by sequence homology, our results suggest that less than 2% of these show promise to be biologically-involved in lncRNA sponging of miRNAs in lung cancer. Thus, our study calls for caution when examining potential miRNA-lncRNA sponging interaction networks. While sequence homology alone is not enough to predict function, the feasibility of these complex gene expression networks is strengthened by considering the context in which these interactions take place. Our study of *XIST*:miRNA:DMX genes in lung cancer provide the groundwork to identify the most biologically-relevant lncRNA sponge targets, an approach that is applicable to other lncRNAs in other pathologies.

## Conclusions

An increasing number of studies have suggested that *XIST* may function as an oncogene through a purported role as a miRNA sponge. While miRNA-*XIST* interaction can occur under engineered and predicted conditions, a true *XIST*-miRNA-DMX scenario will be highly dependent on biological context.

Here, we take a biology-driven approach to identify genes defended from miRNA-based inhibition by the lncRNA *XIST* in lung adenocarcinoma. In the case of *XIST*, we find that important biological considerations include: differences in sex based expression, transcript localization, and tissue specific expression. Additionally, our results suggest that target mRNAs may be mediated through effects of multiple miRNAs rather than one specific miRNA-coding-gene interaction, and that miRNAs that bind to exonic regions of *XIST* exhibit stronger correlations with DMX genes. Furthermore, we predict a set of 124 genes that share 804 miRNAs with *XIST*. We then use the biological properties of *XIST* to identify 13 high-confidence miRNA-DMX pairs. We find that several of these miRNAs preferentially localize to the nucleus (where *XIST* is exclusively located), allowing for the sponging of these species.

Although the sex-specific nature of *XIST* expression provided us with the opportunity to address sponging ability, consideration of biological features such as tissue-specific expression and co-localization should be considered in all lncRNA:miRNA studies. While questions remain as to how widespread the miRNA sponging phenomenon occurs, our study delineates this mechanism in lung adenocarcinoma with regards to *XIST*.

## Methods

### Data processing

*RNA sequencing data*. RNA sequencing data from 568 LUAD and non-malignant samples (304 female and 264 male) were downloaded from CancerBrowser (Illumina HiSeq, https://genome-cancer.ucsc.edu/proj/site/hgHeatmap/). Raw sequencing reads were aligned to the hg19 build of the human genome and quantified against the Ensembl reference gene annotations (Release 75).

*Small RNA sequencing data*. Small RNA sequencing data for the same LUAD samples analyzed above were downloaded from The Cancer Genome Atlas (TCGA). Raw sequencing reads were aligned to the hg19 build of the human genome and quantified relative to miRBase v22 (http://www.mirbase.org/ftp.shtml). MiRNAs with >1 RPKM across 10% of LUAD samples (malignant or non-malignant) were considered for further analysis.

### Data analysis

*Candidate DMX gene identification*. Gene expression analysis was performed in female (n = 274) and male (n = 235) LUAD tumours separately. Spearman’s correlations (Rho) of all Ensembl-annotated genes were performed against *XIST*. P-values were corrected using the Benjimini-Hochberg (B-H) method, and candidate DMX genes were identified as those with Rho>0.4 and B-H p<0.05 in female LUAD tumours (n = 543 genes).

*miRNA binding prediction algorithm*. 3’UTR sequences of all candidate DMX genes were run through the miRanda binding prediction algorithm [22]. Briefly, miRanda uses sequence homology of miRNA and 3’UTR sequences, in addition to binding energies, to predict strength of miRNA binding. Candidate miRNA were selected if they exhibited a net binding energy (ΔG) of at least -20 kCal/mol and a minimum binding score of 150 in both *XIST* and at least one DMX gene 3’UTR. This score and energy threshold was optimized to include perfect complementarily between miRNA and target gene, but be dominated by imperfectly-bound gene pairs. From these candidates, miRNAs were selected as sponging targets if they exhibited a change in correlation (ΔRho) of 0.1 or greater between the male and female cohorts (n = 9 miRNA). All identified candidates were expressed in lung tissue (RPKM>1 in 10% of samples).

### Subcellular fractionation in lung cancer cell lines

*Cell culture*. Cell lines (H1395, H2009, and H2122) were obtained from American Type Culture Collection (ATCC, Manassas, VA, USA) and maintained in RPMI 1640 supplemented with 10% FBS according to ATCC guidelines (Gibco–ThermoFisher, Waltham, MA, USA). Cells were grown in a humidified incubator at 37ᵒC and 5% CO_2_. Cytoplasmic and nuclear fractionation was performed according to published protocols (Abcam, http://www.abcam.com/protocols/subcellular-fractionation-protocol). Briefly, cells were suspended in fractionation buffer (20 mM HEPES, pH 7.4; 10 mM KCl, 2mM MgCl_2_, 1 mM EDTA, 1 mM EGTA). Cells were passed through a 27-gauge needle 12 times for cellular membrane lysis and incubated on ice for 10 minutes. Nuclei were pelleted out of solution, and the remaining pellet was washed with PBS. The nuclear pellet was then resuspended in fractionation buffer, passed through a 25-gauge needle 12 times, and centrifuged for 10 minutes. The remaining nuclear pellet and cytoplasmic fractions were resuspended and split into 2 aliquots for protein and RNA lysate harvesting.

*Western blotting*. Protein was extracted from fractions by resuspeding pellet in RIPA lysis buffer on ice (20 mM Tris-HCl, pH 7.5; 150 mM NaCl; 0.5% DOC, 1% NP-40 and 0.1% SDS), supplemented with protease inhibitors (Complete protease inhibitor cocktail, Roche Diagnostics, Laval, QC, Canada). Protein concentrations were quantified by BCA assay (Pierce, Rockford, IL, USA) according to recommended protocols. Equivalent amounts of total protein were prepared and run on an SDS gel and transferred to a PVDF membrane (Bio-Rad Laboratories, Mississauga, ON, Canada). Membranes were then probed for nuclear and cytoplasmic proteins, Histone H3 (Cell Signaling 9715L) and GAPDH (Cell Signaling 2118L), respectively. Membranes were then probed for HRP-conjugated secondary anti-Rabbit (Cell Signaling 7074S).

*Transcript quantification by RT-qPCR*. RNA was harvested from suspended nuclear and cytoplasmic fractions by resuspending the lysate in 1mL Trizol Reagant. RNA was extracted according to standard Trizol protocols and resuspended in DEPC-treated H_2_O. Relative expression of mRNA and miRNA were quantified using appropriate RT-qPCR systems (TaqMan—Applied Biosystems, Carlsbad, CA). *XIST* (Hs00300535_s1) levels were quantified relative to 18S as an endogenous control (Hs99999901_s1). miRNAs 29b (assay ID 000413), 29a (assay ID 002112), 106a (assay ID 002170), and 1254 (assay ID 002818) were quantified relative to the ratio between the sum of mature and immature miR-29b ratios in both fractions (Cat# 4427975). Custom primers for the immature (pre-miRNA and pri-miRNA) miR-29b, 29a, 106a, and 1254 sequences were designed using the TaqMan system (S6 Table). To differentiate signals from mature and immature miRNA transcripts, the amplification cycle of primers specific to only the pre- and pri-miRNA transcripts were substracted from those from the mature miRNA sequence.

## Supporting information

S1 Fig A) Peer-reviewed publications per year from 2012–2017 on miRNA and *XIST*, as well as two other well-studied lncRNAs; MALAT1, and NEAT1, exhibit a near exponential increase in number (*XIST*: y = 11.8e^0.328x^, R^2^ = 0.952; MALAT1: y = 7.1e^0.553x^, R^2^ = 0.998; NEAT1: y = 2.2e^0.614x^, R^2^ = 0.989).
(TIF)

S2 Fig *XIST* exhibits a wide range of expression in normal lung tissue. Data source: dbGaP Accession phs000424.v7.p2 (ENSG00000229807.5). Expression is presented in transcripts per million (TPM).
(TIF)

S3 Fig miRNA binding sites are distributed throughout introns and exons on the *XIST* transcript. (TIF)

S4 Fig Expression of *XIST* splice isoforms across normal tissues. Fully-spliced *XIST* isoform is displayed in red. Data source: dbGaP Accession phs000424.v7.p2. Expression is shown in transcripts per million (TPM).
(TIF)

S5 Fig Characterization of miRNA binding to *XIST*. A) *XIST* (red) is not enriched in the frequency of miRNA binding sites compared to all other annotated lncRNAs. B) Each predicted miRNA has between 1 and 18 predicted binding sites on the *XIST* transcript. C) Representative images of select candidate DMX genes and their Spearman`s correlation to *XIST* expression. D) Spearman’s correlations between *XIST* and DMX geens are affected by the number of shared miRNA E) DMX genes with more miRNA binding sites are correlated more strongly with *XIST* expression in LUAD females (Top and bottom 15% of total miRNA bindings sites on DMX). Student’s t-test, p = 0.0046).
(TIF)

S6 Fig Gene expression from the Y chromosome is equivalent in High *XIST* and Low *XIST* males. A) Comparison of average normalized reads (RPKM) per patients from the Y chromosome between females, High *XIST* males, and Low *XIST* males. B). Female miRNA-DMX relationships corresponding to the same genes observed in Low and High *XIST* males are equivalent. Student’s t-test, ****: p<0.0001.
(TIF)

S7 Fig Logo of hexanucleotide localization sequence of proposed miRNA enriched in the nucleus. The established miRNA-29b logo (grey) compared to the nucleotide enrichment of the 13 miRNA species proposed to be enriched in the nucleus. miRNAs did not show enrichment of the established sequence at any base.
(TIF)

S1 Table Search terms input into PubMed Central used to generate S1A Fig. (XLSX)

S2 Table miRNA binding position on *XIST* transcript (miRanda binding prediction). (XLS)

S3 Table miRNA binding prediction to DMX genes and *XIST*. (XLSX)

S4 Table Results from Students T-Test of DMX gene expression between male and female LUAD. (XLSX)

S5 Table Exonic and intronic boundaries on *XIST* transcript. (XLSX)

S6 Table Sequences of custom TaqMan small RNA primers. (XLS)

## Abbreviations

ATCC: 1American Type Culture Collection
B-H: Benjimini-Hochberg
DMX genes: Defended from miRNA by *XIST* genes
lncRNA: Long non-coding RNA
LUAD: Lung adenocarcinoma
miRNA: MicroRNA
RT-qPCR: Reverse Transcription Quantitative Polymerase Chain Reaction
TCGA: The Cancer Genome Atlas
UTR: Untranslated region
*XIST*: *X inactive-specific transcript*
