# Supplementary figures and images for: Beyond sequence homology: Cellular biology limits the potential of XIST to act as a miRNA sponge
Source: PLoS One. 2019 Aug 16;14(8):e0221371. doi: 10.1371/journal.pone.0221371 (PMC6697314; doi:10.1371/journal.pone.0221371)

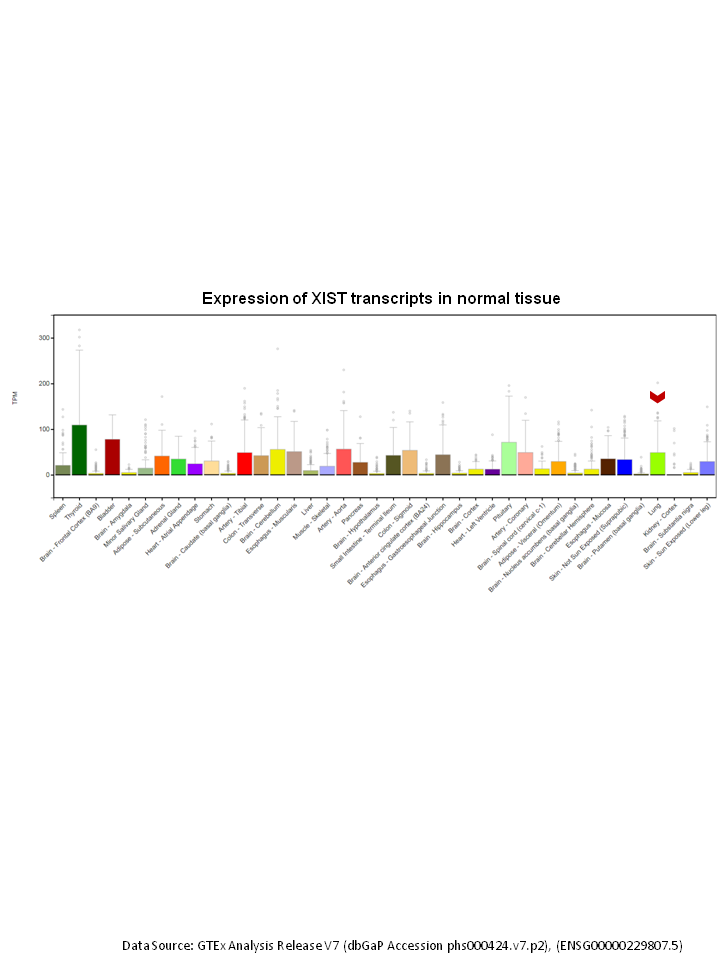

Supplement: S2 Fig — Data source: dbGaP Accession phs000424.v7.p2 (ENSG00000229807.5). Expression is presented in transcripts per million (TPM). (TIF) [file pone.0221371.s002.TIF]

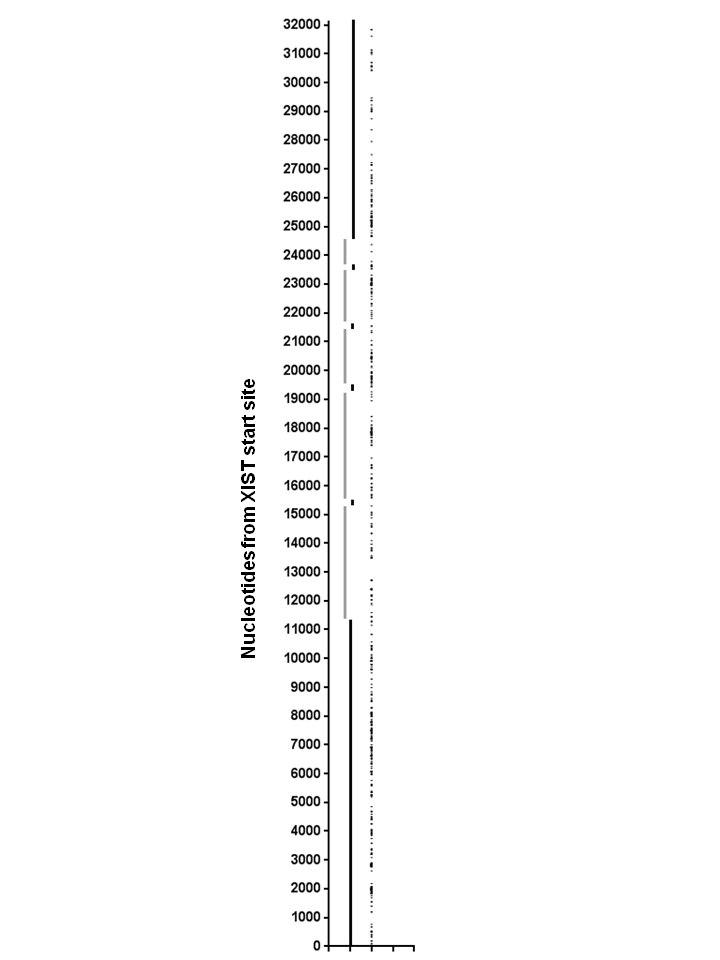

Supplement: S3 Fig — (TIF) [file pone.0221371.s003.TIF]

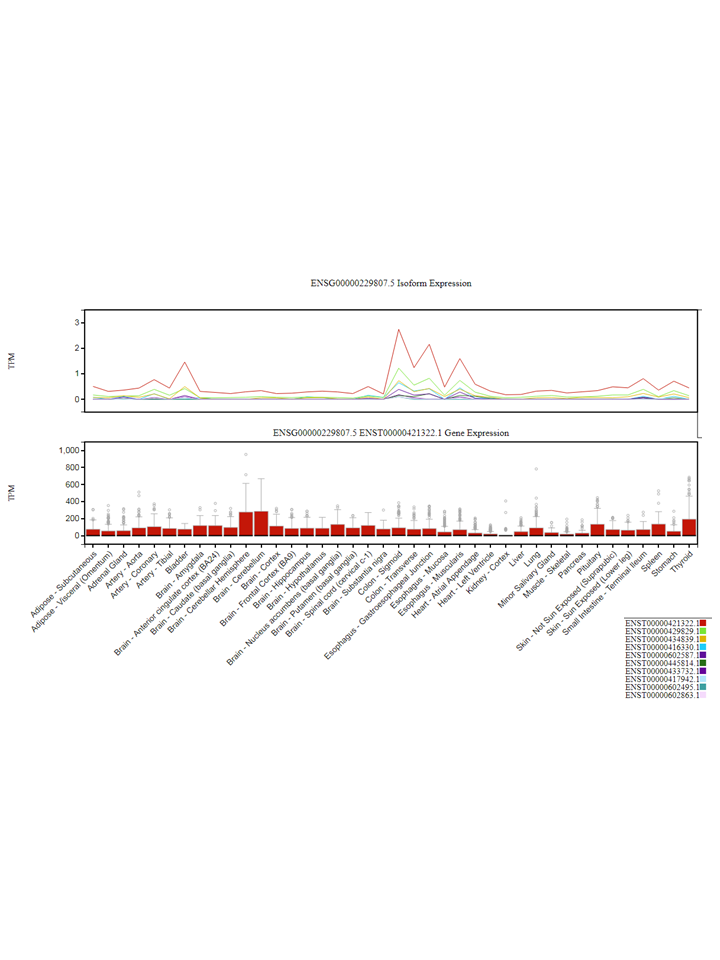

Supplement: S4 Fig — Fully-spliced XIST isoform is displayed in red. Data source: dbGaP Accession phs000424.v7.p2. Expression is shown in transcripts per million (TPM). (TIF) [file pone.0221371.s004.TIF]

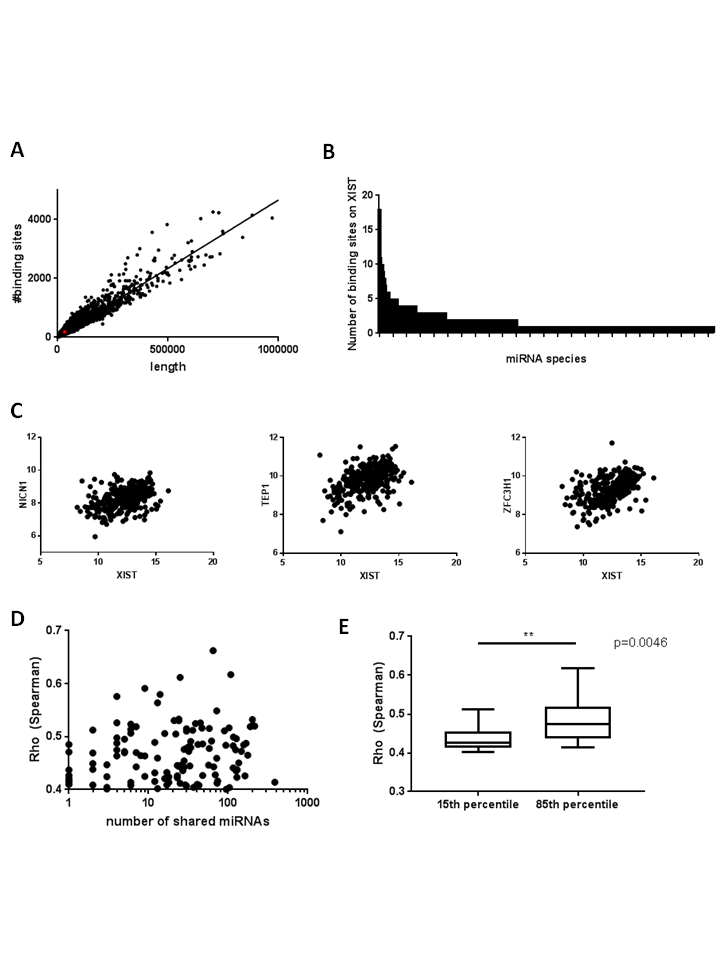

Supplement: S5 Fig — A) XIST (red) is not enriched in the frequency of miRNA binding sites compared to all other annotated lncRNAs. B) Each predicted miRNA has between 1 and 18 predicted binding sites on the XIST transcript. C) Representative images of select candidate DMX genes and their Spearman`s correlation to XIST expression. D) Spearman’s correlations between XIST and DMX geens are affected by the number of shared miRNA E) DMX genes with more miRNA binding sites are correlated more strongly with XIST expression in LUAD females (Top and bottom 15% of total miRNA bindings sites on DMX). Student’s t-test, p = 0.0046). (TIF) [file pone.0221371.s005.TIF]

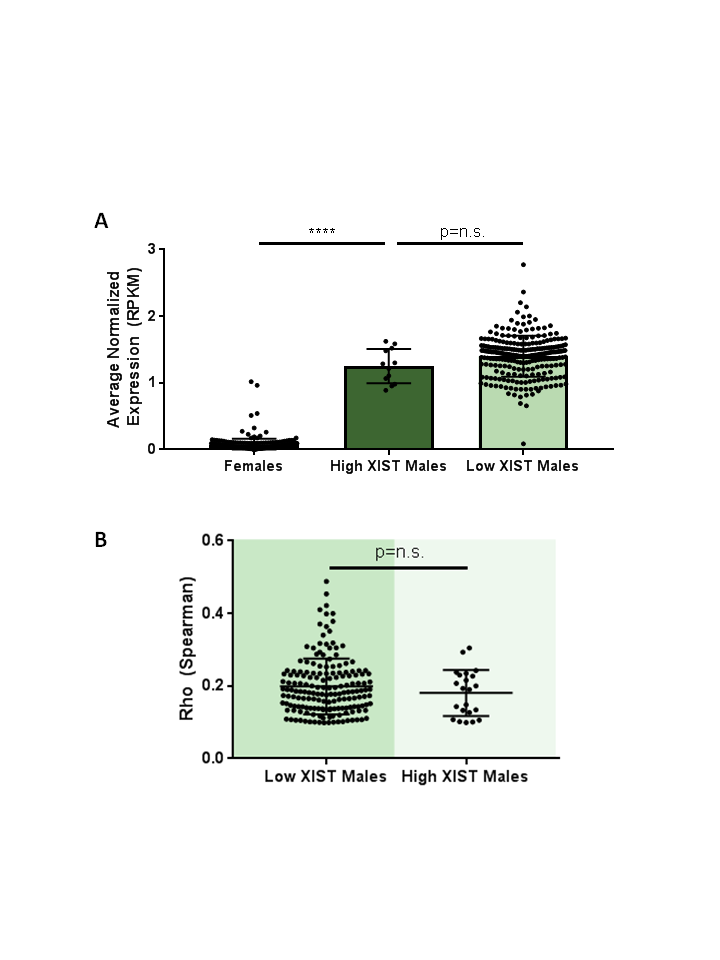

Supplement: S6 Fig — A) Comparison of average normalized reads (RPKM) per patients from the Y chromosome between females, High XIST males, and Low XIST males. B). Female miRNA-DMX relationships corresponding to the same genes observed in Low and High XIST males are equivalent. Student’s t-test, ****: p<0.0001. (TIF) [file pone.0221371.s006.TIF]

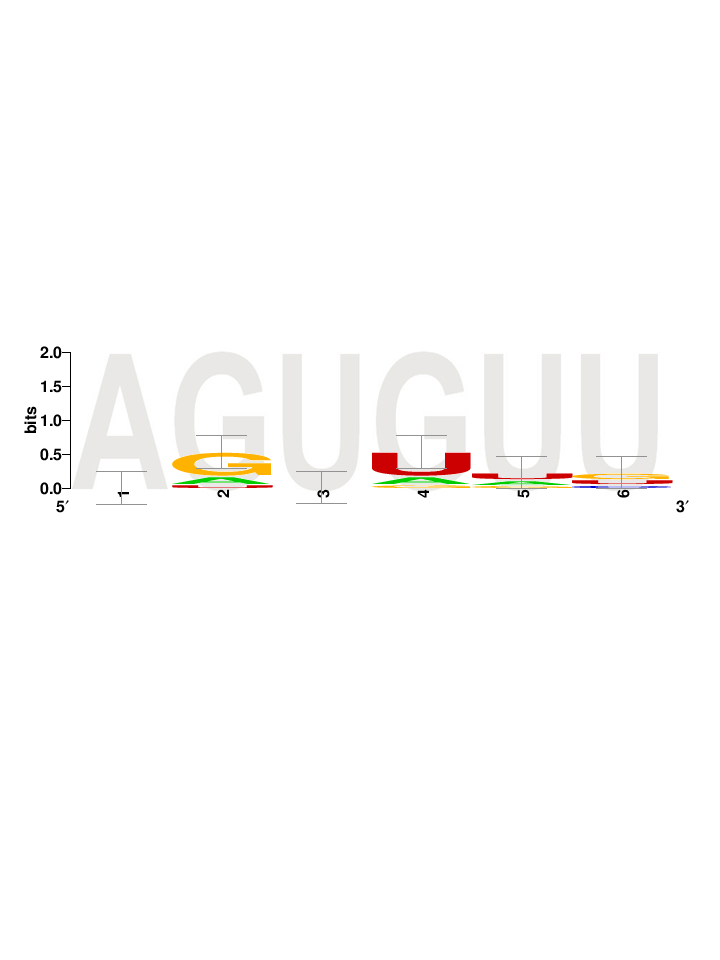

Supplement: S7 Fig — The established miRNA-29b logo (grey) compared to the nucleotide enrichment of the 13 miRNA species proposed to be enriched in the nucleus. miRNAs did not show enrichment of the established sequence at any base. (TIF) [file pone.0221371.s007.TIF]
